# *Clematis pedunculata* (Ranunculaceae), a new species from Korea previously confused with *C. fusca* var. *violacea*

**DOI:** 10.3897/phytokeys.280.213893

**Published:** 2026-10-13

**Authors:** Beom Kyun Park, Dong-Hyuk Lee, Sang-Chul Kim, Dong Chan Son

**Affiliations:** 1 DMZ Forest Biological Resources Research Division, Korea National Arboretum, Yanggu 24564, Republic of Korea DMZ Forest Biological Resources Research Division, Korea National Arboretum Yanggu Republic of Korea; 2 Forest Biodiversity Conservation Research Division, Korea National Arboretum, Pocheon 11186, Republic of Korea Forest Biodiversity Conservation Research Division, Korea National Arboretum Pocheon Republic of Korea; 3 Biodiversity Conservation Research Division, National Institute of Biological Resources, Incheon 22689, Republic of Korea Biodiversity Conservation Research Division, National Institute of Biological Resources Incheon Republic of Korea; 4 Forest Biodiversity Research Division, Korea National Arboretum, Pocheon 11186, Republic of Korea Forest Biodiversity Research Division, Korea National Arboretum Pocheon Republic of Korea

**Keywords:** *Clematis fusca* complex, endemic species, molecular phylogeny, new species, Ranunculaceae, taxonomy

## Abstract

During a taxonomic revision of *Clematis* in Korea, Korean plants previously assigned to *C. fusca* var. *violacea* Maxim. were found to comprise two morphologically distinct groups. To assess the taxonomic significance of this variation, living plants and 452 herbarium specimens were examined, and maximum-likelihood phylogenetic analyses were performed based on sequences from the nuclear ribosomal internal transcribed spacer (ITS), the plastid *matK* gene, and the *atpB–rbcL* intergenic spacer. One of these groups is described here as *Clematis pedunculata* B. K. Park, **sp. nov**. The new species is distinguished from other Korean members of the *C. fusca* complex by a combination of an androdioecious sexual system, smooth to shallowly grooved stem internodes that are sparsely villous to glabrate, conspicuous peduncles 1.5–12.1 cm long, and stamens bearing short whitish hairs. In the ITS phylogeny, the three accessions of *C. pedunculata* formed a moderately supported clade distinct from the sampled Korean members of the *C. fusca* complex, although their deeper phylogenetic placement was weakly resolved. In the combined plastid phylogeny, they formed a distinct clade with moderate support, separate from all sampled Korean members of the complex. The concordant morphological and molecular evidence supports the recognition of *C. pedunculata* as a distinct species currently known only from South Korea.

## Introduction

The genus *Clematis* L. (Ranunculaceae) comprises approximately 300 species distributed mainly in temperate regions. Its members are predominantly herbaceous or woody climbers characterized by apetalous flowers and achenes bearing persistent, often plumose styles (Tamura 1967, 1987, 1995; Wang and Bartholomew 2001; Kadota 2006; Kim 2017). Despite extensive morphological and molecular studies, the circumscription and phylogenetic relationships of several morphologically variable species complexes within the genus remain unresolved (Miikeda et al. 2006; Xie et al. 2011; Lehtonen et al. 2016). Anatomical and micromorphological characters of petioles and achenes have also provided useful evidence for delimiting Korean *Clematis* taxa (Ghimire et al. 2020; Park et al. 2021).

Thirteen taxa of *Clematis* are recognized in South Korea (Kim 2017). Recent taxonomic studies have continued to reveal previously unrecognized diversity among Korean members of the genus (Kim et al. 2009; Park et al. 2022). Within the Korean flora, the *C. fusca* complex has generally been treated as comprising *C. fusca* var. fusca, *C. fusca* var. *flabellata*, and *C. fusca* var. *violacea* (Lee 1996; Chang 2007; Kim 2017). Although morphologically similar, these three taxa can be distinguished by differences in growth form, stem morphology, peduncle development, and the indumentum of the sepals and stamens (Lee 1996; Chang 2007; Kim 2017).

The taxonomic identity of Korean plants assigned to *C. fusca* var. *violacea* has remained uncertain. The protologue of the variety emphasized violet sepals with whitish hairs largely confined to their margins (Maximowicz 1859), and subsequent Korean floristic treatments broadly applied the name to plants sharing this character combination (Lee 1996; Lee 2003; Lee 2006; Chang 2007; Kim 2017). Preliminary field observations and examination of herbarium specimens indicated the presence of two morphologically coherent groups among Korean collections identified under this name. These groups differ consistently in floral sexuality, peduncle development, the morphology and indumentum of the stem internodes, and the indumentum of the sepals and stamens, indicating that the observed variation may have taxonomic significance. Accordingly, Korean plants assigned to *C. fusca* var. *violacea* were reassessed to determine whether the two morphological groups represent intraspecific variation or distinct taxonomic entities and to clarify their relationships to other members of the Korean *C. fusca* complex.

## Material and methods

### Morphological analysis

Eleven freshly collected specimens representing a putatively distinct morphological group within the *Clematis fusca* complex and three related taxa were examined, and voucher specimens were deposited in the herbarium of the Korea National Arboretum (KH). Field photographs were taken using digital cameras (Nikon D3X, D7200, and COOLPIX P530; Nikon Corporation, Tokyo, Japan). In addition, 452 herbarium specimens belonging to the *C. fusca* complex, including specimens representing the putative new taxon housed at KH, were examined (Appendix 1). Digital images of the type specimens and other relevant specimens of *C. fusca* var. fusca, *C. fusca* var. *flabellata*, and *C. fusca* var. *violacea* housed at the Komarov Botanical Institute of the Russian Academy of Sciences (LE) and the Royal Botanic Garden Edinburgh (E) were also examined. Taxonomic identifications and morphological comparisons were based on the relevant protologues—particularly that of *C. fusca* var. *violacea* (Maximowicz 1859)—and on regional floras and taxonomic monographs (Wang and Bartholomew 2001; Kadota 2006; Kim 2017).

Morphological measurements were taken using digital calipers (Mitutoyo CD-15APX; Mitutoyo Corporation, Kawasaki, Japan), and vegetative and reproductive structures were examined under a stereomicroscope (Olympus SZX16; Olympus Corporation, Tokyo, Japan). Floral structures were dissected as necessary. The characters assessed included leaflet number and size, petiole morphology, and the size, shape, and indumentum of the bracts, sepals, stamens, and carpels (Table 1). Herbarium acronyms follow the continuously updated Index Herbariorum (2026).

**Table 1.** Comparison of morphological characters of *Clematis pedunculata* and related taxa.

	***C. fusca* var. fusca**	***C. fusca* var. *flabellata***	***C. fusca* var. *violacea***	***Clematis pedunculata***
**Habit**	Woody vine	Subshrub	Herbaceous vine	Herbaceous vine
**Growing type**	Scandent	Erect	Scandent	Scandent
**Stem node trichome**	Densely villous	Densely villous	Villous	Villous
**Stem internode trichome**	Villous	Villous	White villous	Sparsely villous and glabrate
**Stem groove**	Distinctly grooved	Distinctly grooved	Distinctly grooved	Smooth to indistinctly grooved
**Petiole type**	Tendril-like	Petiolate	Tendril-like	Tendril-like
**Petiolule type**	Tendril-like	Petiolate	Tendril-like	Tendril-like
**Petiolule trichome**	Sparsely pubescent	Villous	sparsely villous	Sparsely villous
**Leaflet terminal type**	Mostly degenerated	Generated	Mostly degenerated	Mostly degenerated
**Leaflet terminal shape**	Ovate-lanceolate to widely ovate	Ovate-lanceolate to widely ovate	Ovate-lanceolate to widely ovate	Ovate to widely ovate
**Leaflet terminal length (cm)**	1.3 (6.8) 11.2	2.3 (5.1) 11.3	1.1 (3.3) 7.3	1.3 (6.3) 10.8
**Leaflet terminal width (cm)**	1.1 (3.2) 5.2	1.3 (3.5) 6.4	0.5 (3.1) 6.2	1.8 (3.4) 5.1
**Leaflet lateral shape**	Ovate-lanceolate to widely ovate	Ovate-lanceolate to widely ovate	Ovate-lanceolate to widely ovate	Ovate to widely ovate
**Leaflet lateral length (cm)**	4.2 (6.7) 8.5	2.2 (5.8) 9.8	3.2 (6.7) 9.6	2.9 (7.8) 11.2
**Leaflet lateral width (cm)**	1.9 (4.2) 6.5	1.2 (4.8) 7.2	2.1 (5.2) 8.2	2.5 (7.6) 15.2
**Leaflet adaxial surface**	Sparsely pubescent	Pubescent	Sparsely pubescent	Sparsely pubescent
**Leaflet abaxial surface**	Villous and pubescent	Sparsely villous	Sparsely pubescent	Villous and pubescent
**Inflorescences type**	Cyme	Solitary	Cyme	Cyme
**Inflorescences position**	Terminal and axillary	Terminal	Terminal and axillary	Terminal and axillary
**Inflorescences flower number**	1 or 3	1	1 or 3	1 or 3
**Bract number**	Two or terminal flower absent	Absent	Two or terminal flower absent	Two or terminal flower absent
**Bract shape**	Lanceolate or widely ovate	Absent	Lanceolate or widely ovate	Lanceolate or widely ovate
**Bract length (mm)**	9.7 (21.9) 33.4	Absent	8.7 (16.4) 22.6	16.3 (26.4) 53.9
**Bract width (mm)**	7.2 (11.9) 16.4	Absent	6.5 (13.8) 16.1	10.4 (22.6) 60.1
**Bract margin**	Entire to 3-lobed	Absent	Entire to 3-lobed	Entire to 3-lobed
**Bract trichome**	Pubescent	Absent	Pubescent	Pubescent
**Peduncle presence**	Subsessile or terminal flower absent	Absent	Subsessile or terminal flower absent	Pedunculate or terminal flower absent
**Peduncle length (mm)**	1.1 (2.3) 6.2	Absent	0.9 (3.4) 6.7	14.7 (65.4) 121.1
**Peduncle trichome**	Densely blackish or yellowish brown pubescent	Absent	Densely whitish or yellowish brown pubescent	Sparsely white pubescent
**Pedicel length (cm)**	0.6 (2.2) 3.1	0.8 (2.3) 3.4	0.8 (1.3) 2.2	0.4 (1.9) 2.3
**Pedicel trichome**	Densely blackish brown villose	Densely blackish brown tomentose	Whitish or yellowish brown pubescent	Whitish pubescent
**Flowering season**	Jun to Jul	May to Jun	Jun to Jul	Jun to Jul
**Flower sexuality**	Hermaphroditic	Hermaphroditic	Hermaphroditic	Androdioecious
**Sepal apex**	Acuminate, acute, obtuse or rounded	Acuminate, acute, obtuse or rounded	Acuminate or acute	Acuminate
**Sepal adaxial color**	Purplish brown to blackish purple	Whitish brown to blackish brown	Violetish white to dark purple	Purplish white to reddish purple
**Sepal adaxial trichome**	Sparsely white to dark brown pubescent	Sparsely white to brown pubescent	Sparsely white to yellowish brown pubescent and almost glabrous	Sparsely white pubescent and almost glabrous
**Sepal abaxial color**	White to blackish brown	White to dark brown	Violetish white to purplish white	Purplish white to dark purple
**Sepal abaxial trichome**	Densely blackish brown tomentose	Densely blackish brown tomentose	Whitish to yellowish brown pubescent	Sparsely whitish pubescent and glabrate
**Sepal margin**	Densely blackish brown tomentose	Densely blackish brown tomentose	Densely whitish villous	Densely whitish villous
**Stamen trichome**	Brown villous at upper part	Brown villous at upper part	Brown villous at upper part	White pubescent at upper part
**Anther trichome**	Densely to sparsely brown villous	Densely to sparsely brown villous	Densely to sparsely brown villous	White or yellowish white pubescent

### DNA analysis

#### Taxon sampling

Eleven individuals representing the *C. fusca* complex, including specimens assigned to *C. fusca* var. fusca, *C. fusca* var. *flabellata*, and *C. fusca* var. *violacea*, were sampled for molecular analysis, which revealed the presence of a distinct taxon subsequently identified as *Clematis pedunculata* (Table 2). Fresh leaves were dried in silica gel, and voucher specimens were deposited in KH.

**Table 2.** Collection localities, voucher information, and GenBank accession numbers of newly sequenced samples.

**Taxa**	**Locality**	**Voucher**	**Accession number**		
			**ITS**	***matK***	***atpB-rbcL***
*C. pedunculata*	Korea: Chungcheongbuk-do, Danyang-gun, Yeongcheon-ri	Danyangforest-210706-001	PX342714	PX353127	PX353138
*C. pedunculata*	Korea: Gyeongsangbuk-do, Mungyeong-si, Mawon-ri, Mt. Baekhwa	Baekhwasan-200721-001	PX342716	PX353129	PX353140
*C. pedunculata*	Korea: Gyeongsangbuk-do, Mungyeong-si, Mawon-ri, Mt. Baekhwa	Baekhwasan-210722-001	PX342717	PX353130	PX353141
*C. fusca* var. *violacea*	Korea: Gyeonggi-do, Ansan-si, Sasa-dong, Mt. Chilbo	Chilbosan-210717-001	PX342715	PX353128	PX353139
*C. fusca* var. *violacea*	Korea: Gyeonggi-do, Pocheon-si, Jikdong-ri	Sumokwon-220501-001	PX342718	PX353131	PX353142
*C. fusca* var. *violacea*	Korea: Gyeonggi-do, Pocheon-si, Jikdong-ri	Sumokwon-220711-001	PX342719	PX353132	PX353143
*C. fusca* var. *violacea*	Korea: Gyeonggi-do, Pocheon-si, Jikdong-ri	Sumokwon-200501-001	PX342724	PX353137	PX353148
*C. fusca* var. *fusca*	Korea: Gangwon-do, Hoengseong-gun, Mt. Cheongtae	Cheongtaesan-190719-001	PX342720	PX353133	PX353144
*C. fusca* var. *fusca*	Korea: Gangwon-do, Hoengseong-gun, Mt. Cheongtae	Cheongtaesan-190819-001	PX342721	PX353134	PX353145
*C. fusca* var. *flabellata*	Korea: Gangwon-do, Jeongseon-gun, Mt. Gariwang	Gariwangsan-190529-001	PX342722	PX353135	PX353146
*C. fusca* var. *flabellata*	Korea: Gangwon-do, Jeongseon-gun, Eundaebong	Eundaebong-190530-001	PX342723	PX353136	PX353147

#### DNA extraction, amplification, and sequencing

Genomic DNA was extracted from silica-dried leaves using a DNeasy Plant Mini Kit (Qiagen, Korea) following the manufacturer’s protocol. Nuclear ribosomal ITS was amplified using the primers AB101 and AB102. Plastid *matK* was amplified using the primer pairs 3914F/1470R and C3F/C4R, whereas the *atpB–rbcL* intergenic spacer was amplified using the primers atpBF and rbcLR, following Miikeda et al. (2006).

Each PCR was performed in a final volume of 30 μL containing 10–20 ng of genomic DNA, 1 μL of each primer, and 10 μL of AccuPower PCR PreMix (Bioneer, South Korea), with nuclease-free water added to the volume. The PCR amplification conditions differed depending on the target region. For cpDNA, the profile consisted of an initial denaturation at 94 °C for 1 min; 30 cycles of denaturation at 94 °C for 1 min, annealing at 53 °C for 1 min, and extension at 72 °C for 1 min; and a final extension at 72 °C for 5 min. For ITS, the profile consisted of an initial denaturation at 94 °C for 5 min; 35 cycles of denaturation at 94 °C for 30 s, annealing at 52 °C for 1 min 30 s, and extension at 72 °C for 1 min 30 s; and a final extension at 72 °C for 7 min. PCR products were visualized on 2% agarose gels and sequenced by Macrogen Inc. (Seoul, Korea).

### Phylogenetic analysis

Sequence chromatograms were assembled and edited using Geneious v. 7.1.9 (Biomatters Ltd., New Zealand), and sequences were aligned using MAFFT (Katoh and Standley 2013). The *matK* and *atpB–rbcL* alignments were concatenated to form a combined plastid dataset. Maximum-likelihood analyses were conducted using IQ-TREE (Nguyen et al. 2015) implemented in PhyloSuite (Zhang et al. 2020). The best-fitting substitution model for each dataset was selected using ModelFinder according to the Bayesian information criterion (BIC; Kalyaanamoorthy et al. 2017), resulting in HKY+F+I for the ITS dataset and F81+F+I for the combined plastid dataset. Branch support was assessed using 1,000 ultrafast bootstrap replicates (Minh et al. 2013). Sequences of additional *Clematis* taxa were obtained from GenBank (Table 3), and *Pulsatilla koreana* (Y. Yabe ex Nakai) Nakai ex T. Mori was selected as the outgroup based on previous phylogenetic studies of the tribe Anemoneae (Wang et al. 2009; Jiang et al. 2017).

**Table 3.** GenBank accession numbers of additional taxa and the outgroup used in the phylogenetic analyses.

**Taxa**	**ITS**	***matK***	***atpB-rbcL***
*Clematis baldwinii*	KR909796	KR909810	KR909738
*C. crispa*	GU732589	KR909824	GU732508
*C. fremontii*	KR909792	KR909828	KR909752
*C. fusca* var. *fusca*	AB120179	AB110535	AB115429
*C. nobilis*	AB120206	AB110524	AB115456
*C. ochotensis*	AB120182	AB110501	AB115432
*C. ochroleuca*	KR909793	KR909844	KR909747
*C. serratifolia*	AB120205	AB110523	AB115455
*C. texensis*	AB120197	AB110516	AB115447
*C. viticaulis*	KR909794	KR909857	KR909746
*Pulsatilla koreana*	KF896285	MG952897	MG952897

## Results

### Morphological differentiation

**Figure 1.**
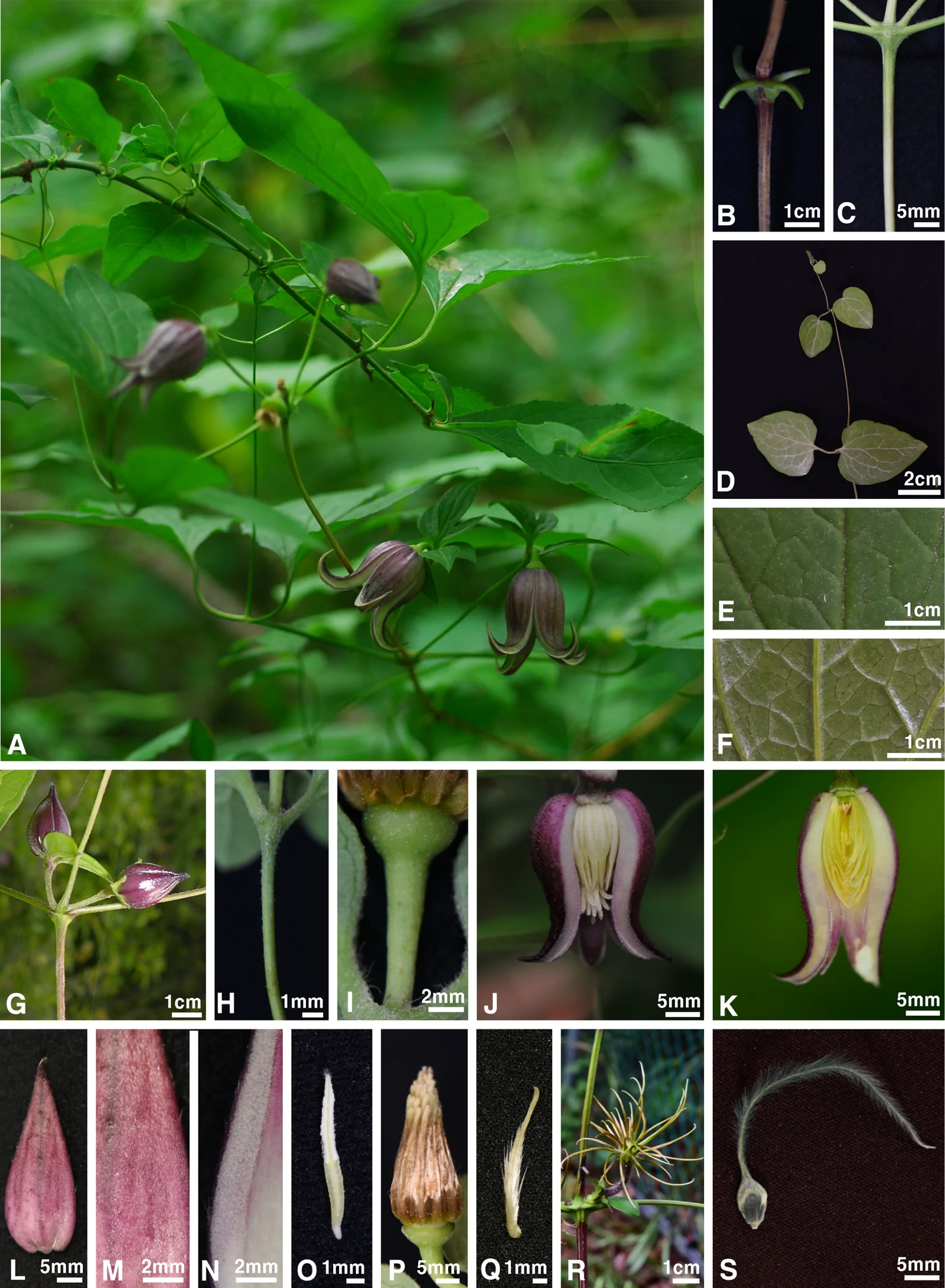
Photographs of *Clematis pedunculata* B.K. Park. **A**. Habit; **B**. Basal portion of stem; **C**. Distal portion of stem; **D–F**. Leaves; **D**. General view of the leaf; **E**. Adaxial surface; **F**. Abaxial surface; **G**. Inflorescence; **H**. Peduncle; **I**. Pedicel; **J**. Bisexual flower; **K**. Staminate flower; **L–N**. Sepals; **L**. General view of the sepal; **M**. Abaxial surface; **N**. Adaxial surface; **O**. Stamen; **P, Q**. Pistils; **R**. Aggregate of achenes; **S**. Achene.

Examination of living plants and herbarium specimens revealed two morphologically coherent groups among Korean collections previously identified as *Clematis fusca* var. *violacea*. The two groups differed consistently in floral sexuality, the morphology and indumentum of the stem internodes, peduncle development, and stamen pubescence. Detailed morphological comparisons and character ranges are provided in Table 1, while the diagnostic features of the newly recognized taxon are illustrated in Fig. 1.

### Phylogenetic analyses

**Figure 2.**
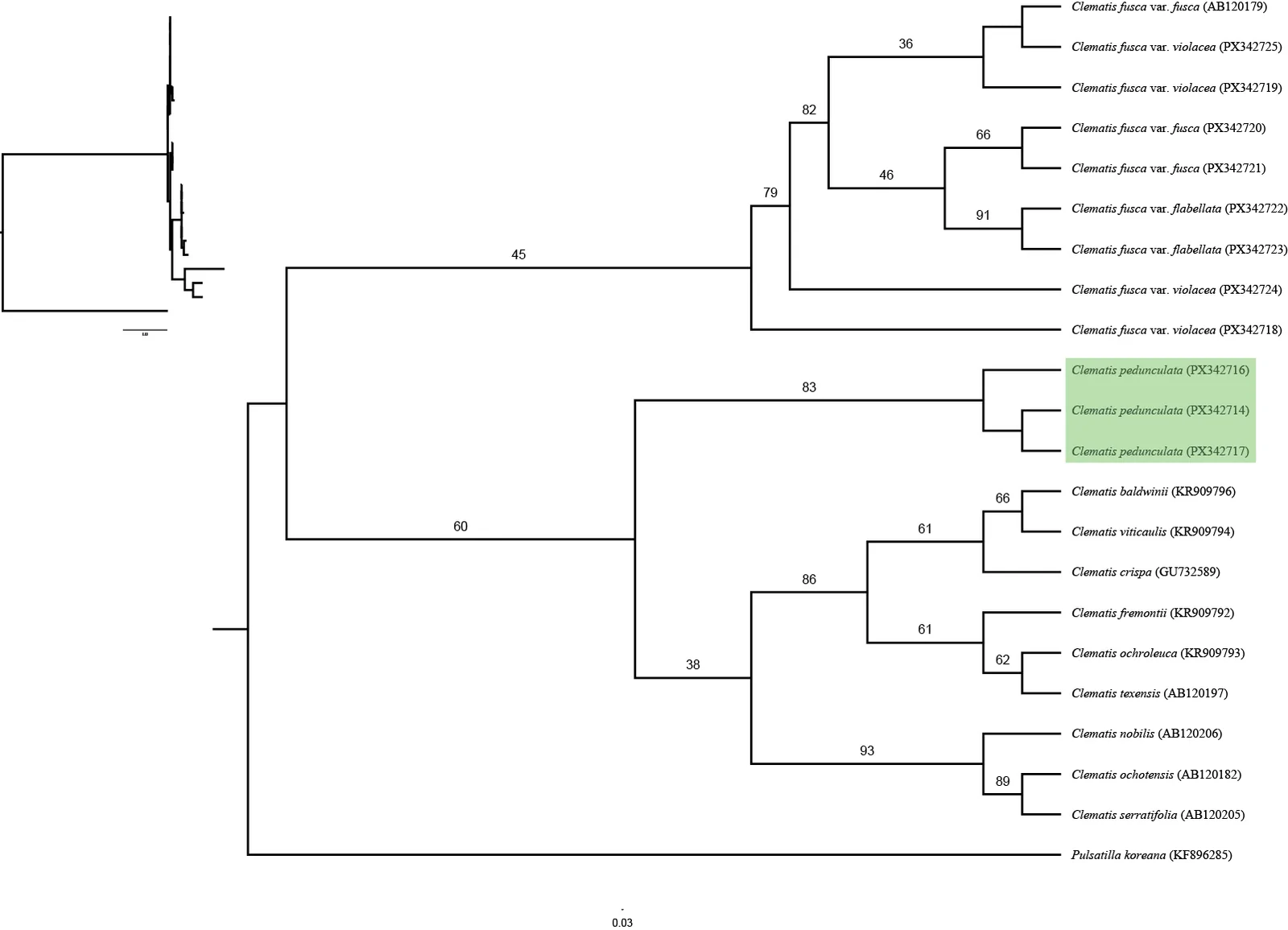
Maximum-likelihood phylogeny inferred from nuclear ribosomal ITS sequences of *Clematis pedunculata* and related taxa. Values at nodes indicate ultrafast bootstrap support.

**Figure 3.**
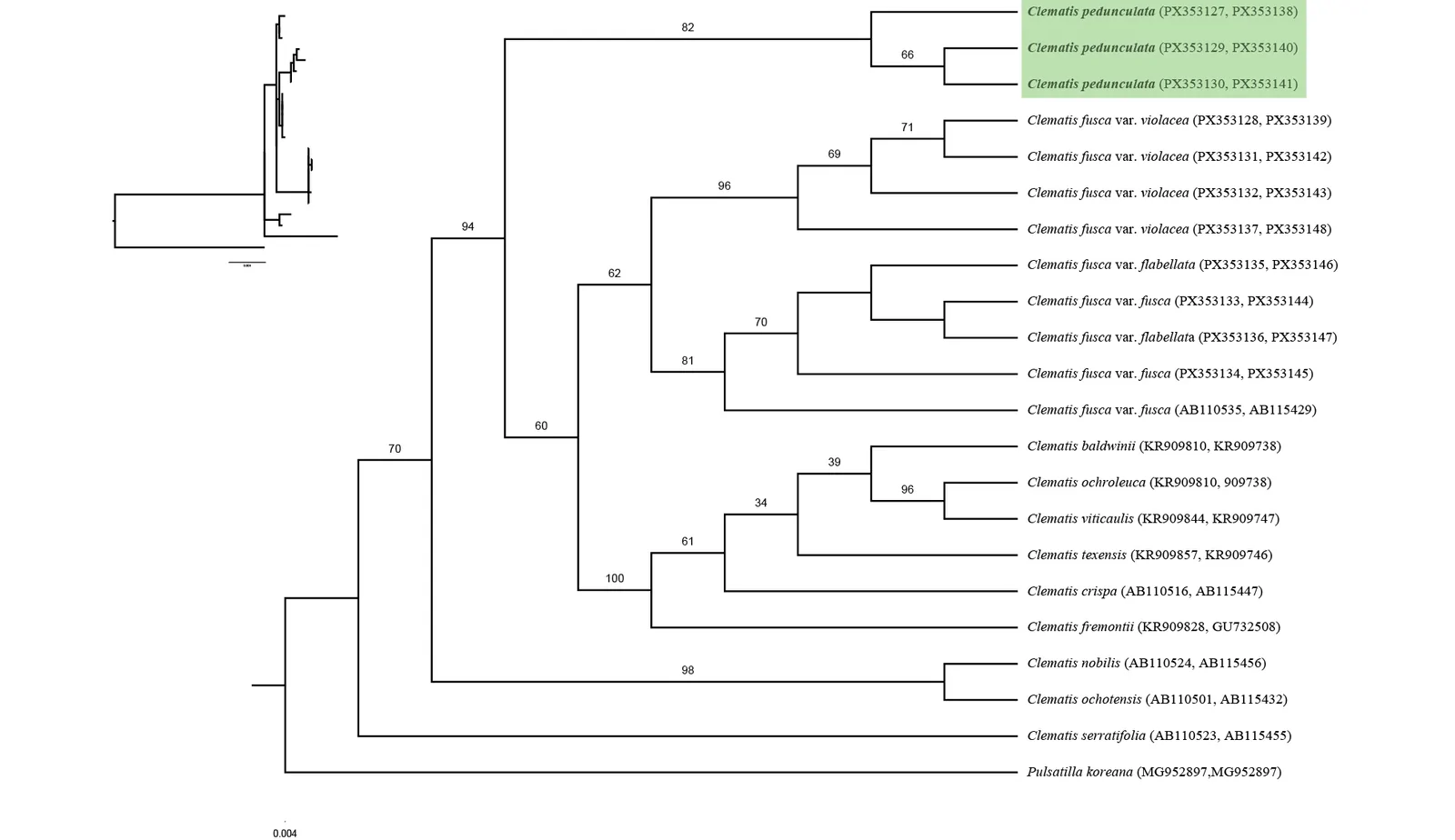
Maximum-likelihood phylogeny inferred from the combined plastid *matK* and *atpB–rbcL* dataset of *Clematis pedunculata* and related taxa. Values at nodes indicate ultrafast bootstrap support.

In the ITS phylogeny, three accessions representing the distinct taxon subsequently identified as *Clematis pedunculata* formed a moderately supported clade (UFBoot = 83%) separate from the sampled Korean members of the *C. fusca* complex; however, the deeper phylogenetic position of this clade was weakly supported (Fig. 2). In the combined plastid phylogeny, the three accessions formed a moderately supported clade (UFBoot = 82%) distinct from all sampled Korean members of the *C. fusca* complex (Fig. 3).

### Taxonomic treatment

#### Clematis pedunculata

Taxon classificationPlantaeRanunculalesRanunculaceae

B.K.Park sp. nov.

FEBCCDDB-BC0B-5A20-8667-0D482B6C0535

urn:lsid:ipni.org:names:77412310-1

##### Type.

**Figure 4.**
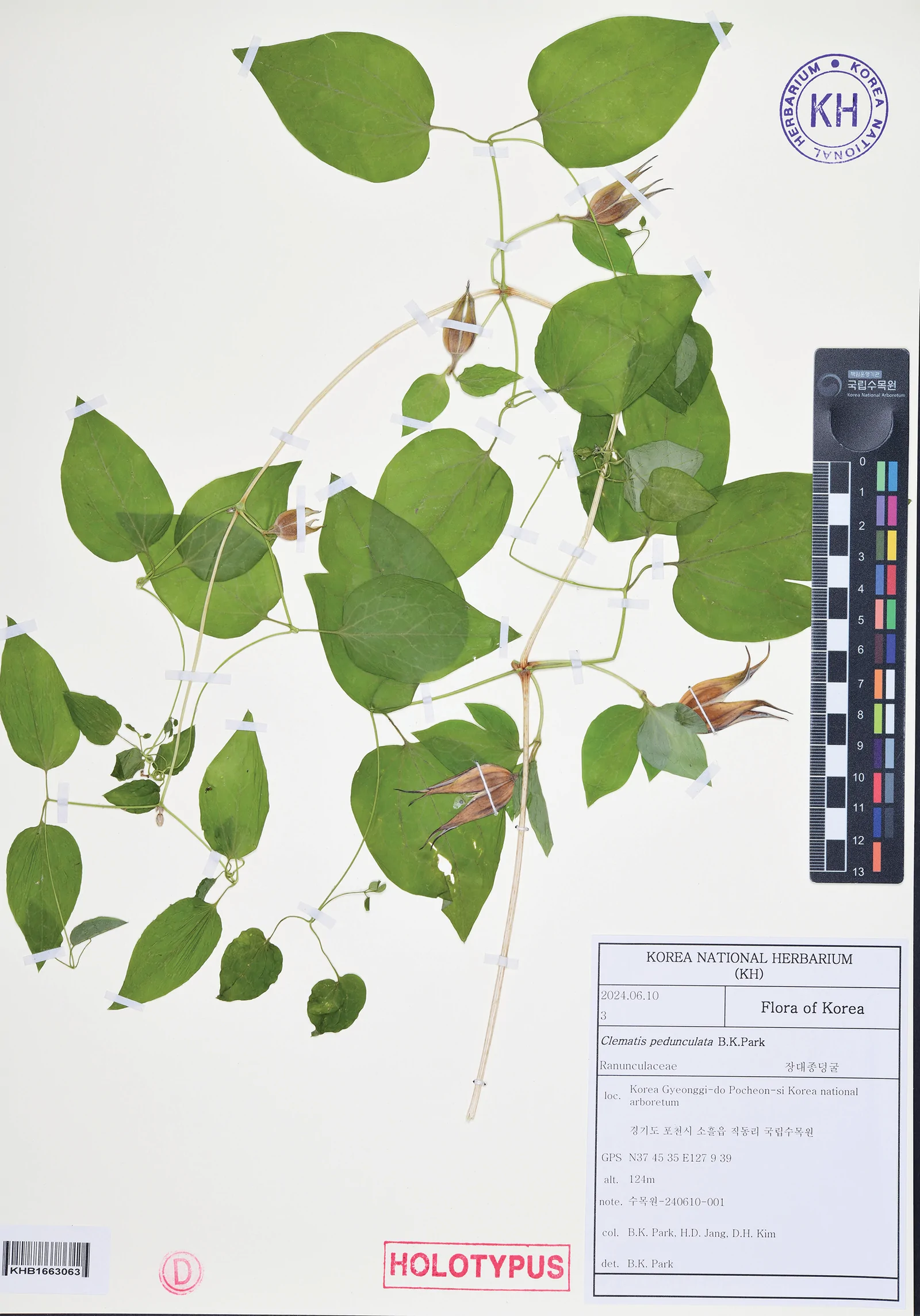
Holotype specimen of *Clematis pedunculata* B.K. Park.

Korea • Gyeonggi-do, Pocheon-si, Soheul-eup, Jikdong-ri, 10 Jun. 2024, *B.K. Park, H.D. Jang and D.H. Kim, Sumokwon-240610-001* [holotype: KH (Fig. 4); isotype, 1 sheet, KH).

##### Diagnosis.

*Clematis pedunculata* is morphologically similar to *C. fusca* var. *violacea* but differs from it in having androdioecious plants (vs. hermaphroditic plants), smooth to shallowly grooved and sparsely villous to glabrate stem internodes (vs. distinctly grooved and villous), conspicuous peduncles 1.5–12.1 cm long (vs. subsessile or 0.9–6.7 mm long), and stamens with short whitish hairs distally (vs. brown-villous distally) (Fig. 1; Table 1).

##### Description.

Perennial herbaceous vines, androdioecious, scandent, reaching ca. 4 m in length. Stems yellowish green to purplish green; nodes 2.9–7.9 mm diameter, villous; internodes 3.9–20.1 cm long, 1.1–3.2 mm diameter, yellowish green to purplish green, smooth or indistinctly grooved, sparsely villous to glabrate. Leaves opposite, simple or 3–7-foliolate, ternate or pinnate; petioles tendril-like, 2.8–8.6 cm long, 0.8–2.5 mm in diameter, yellowish green to reddish green, sparsely villous; petiolules tendril-like, yellowish green to reddish green, sparsely villous, terminal petiolule 0.5–5.1 cm long, 0.7–1.5 mm in diameter, lateral petiolules 0.5–4.3 cm long, 0.8–2.7 mm in diameter; leaflets ovate to widely ovate, apices acute to acuminate, margin entire and lobed, cleft or divided, base acute, rounded or subcordate; adaxial surfaces yellowish green to dark green, sparsely pubescent; abaxial surfaces pale green, villous and pubescent; terminal leaflets 1.3–10.8 × 1.8–5.1 cm; lateral leaflets 2.9–11.2 × 2.5–15.2 cm. Inflorescences cymes, terminal or axillary, 1–3-flowered. Bracts 1-paired or terminal flower absent, petiolate, 1.6–5.4 × 1–6 cm, ovate to widely ovate, entire to 3-lobed, yellowish green to green, pubescent. Peduncle conspicuous, 1.5–12.1 cm long, yellowish green or reddish green, sparsely white pubescent; pedicels 0.4–2.3 cm long, yellowish green or reddish green, whitish pubescent. Flowers staminate or bisexual, nodding, actinomorphic. Sepals 4, 1.2–3.3 cm × 5.3–12.2 mm, ovate to oblong-ovate, apices acuminate, purplish white to purple, adaxial surface sparsely white pubescent and almost glabrous, abaxial surface sparsely pubescent to glabrate, and densely whitish villous along the margins. Stamens numerous; filaments 4.5–12.1 × 0.6–1.4 mm, white to yellowish white, white pubescent at upper part; anthers 3.3–7.1 × 0.5–0.8 mm, linear, lanceolate or oblong, white to pale yellowish white, white to yellowish white pubescent. Pistils numerous and well developed in bisexual flowers, reduced or rudimentary in staminate flowers. Ovaries 0.5–1.6 mm × 0.5–1.1 mm, ovoid or ellipsoid, yellowish green or green, white or yellowish brown villous; styles 6.2–16.6 mm long, green, densely yellowish brown to brown sericeous. Achenes 4.9–6.8 mm × 4–5.9 mm, flattened obovoid or ellipsoid, yellowish green or green, dark brown in maturity, white to yellowish brown villous; persistent styles 3.5–6.6 cm long, plumose, densely yellowish brown to brown sericeous.

##### Phenology.

Flowering from June to July and fruiting from July to September.

##### Etymology.

The specific epithet “*pedunculata*” refers to the presence of distinct peduncles.

##### Korean vernacular name.

Jang-dae-jong-deong-gul (장대종덩굴), proposed here in reference to its conspicuously elongated peduncles.

##### Distribution and habitat.

**Figure 5.**
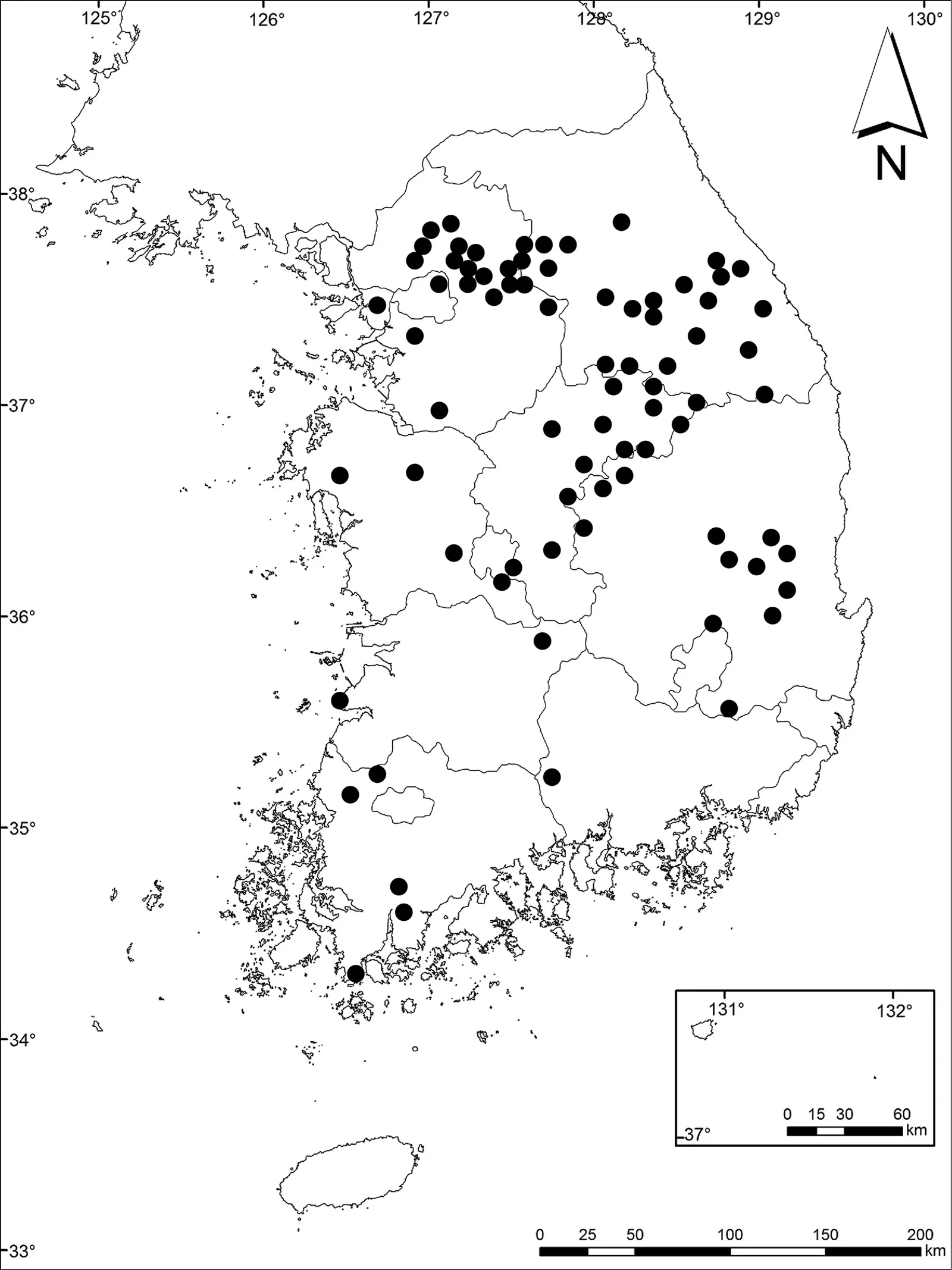
Distribution map of *Clematis pedunculata* B.K. Park.

*Clematis pedunculata* is currently known only from South Korea, where it has been recorded from Gyeonggi-do, Gangwon-do, Chungcheongbuk-do, Chungcheongnam-do, Jeollabuk-do, Jeollanam-do, Gyeongsangbuk-do, and Gyeongsangnam-do (Fig. 5). It occurs in inland forests, along forest margins, and occasionally on roadsides.

#### Clematis fusca

Taxon classificationPlantaeRanunculalesRanunculaceae

var . violacea Maxim., Prim. Fl. Amur. 11, 1859.

F18AA822-85BF-5943-B734-4844008B9961

##### Type.

**Figure 6.**
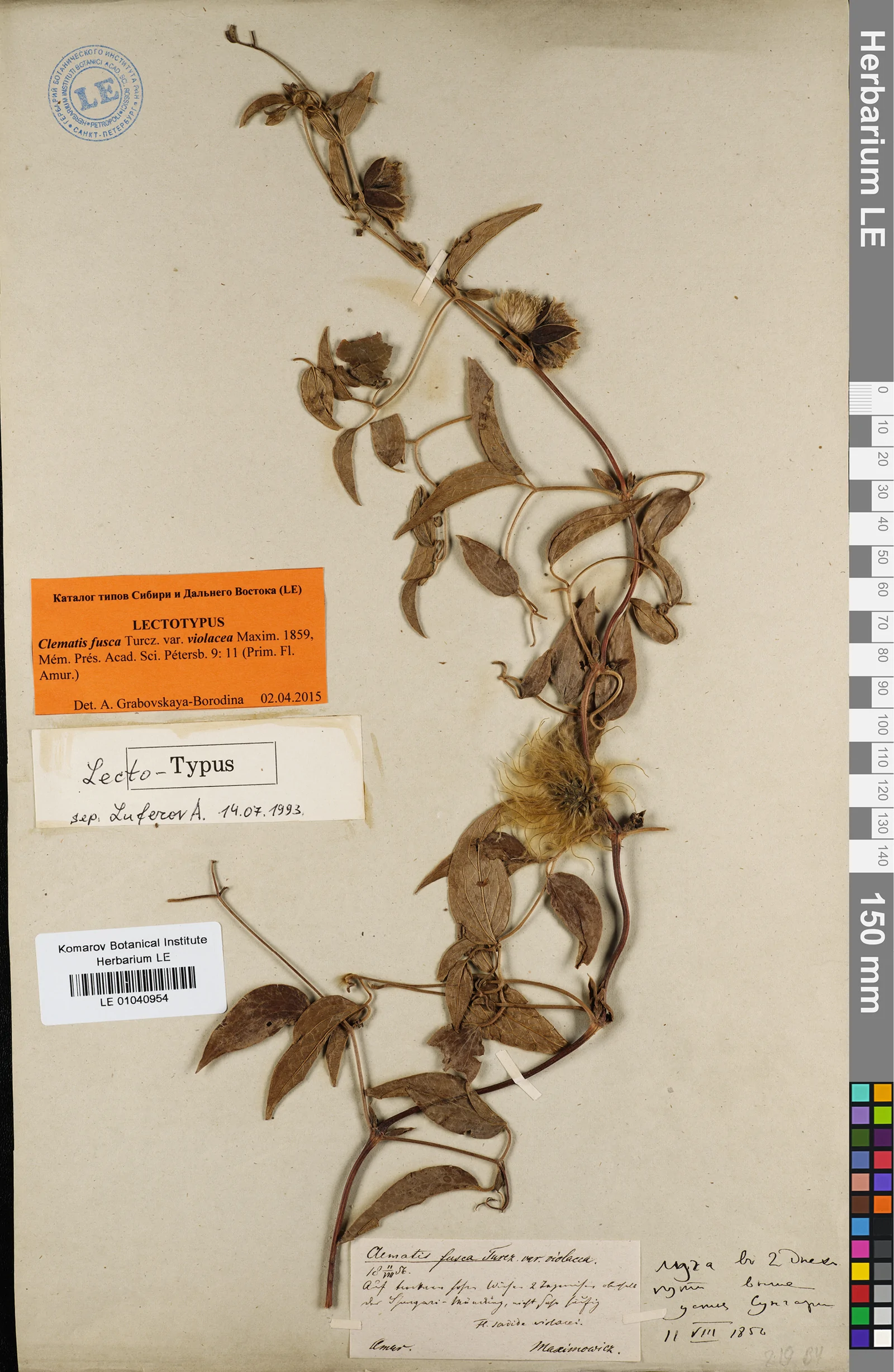
Lectotype specimen of *Clematis fusca* var. *violacea* Maxim.

Russia • Amur oblast: the Sungari River, Meadows 2 days’ journey up from the mouth of the Sungari River, 11 Aug. 1856, *C.J. Maximowicz, s.n*. (lectotype: LE [LE 01040954], Fig. 6).

##### Diagnosis.

**Figure 7.**
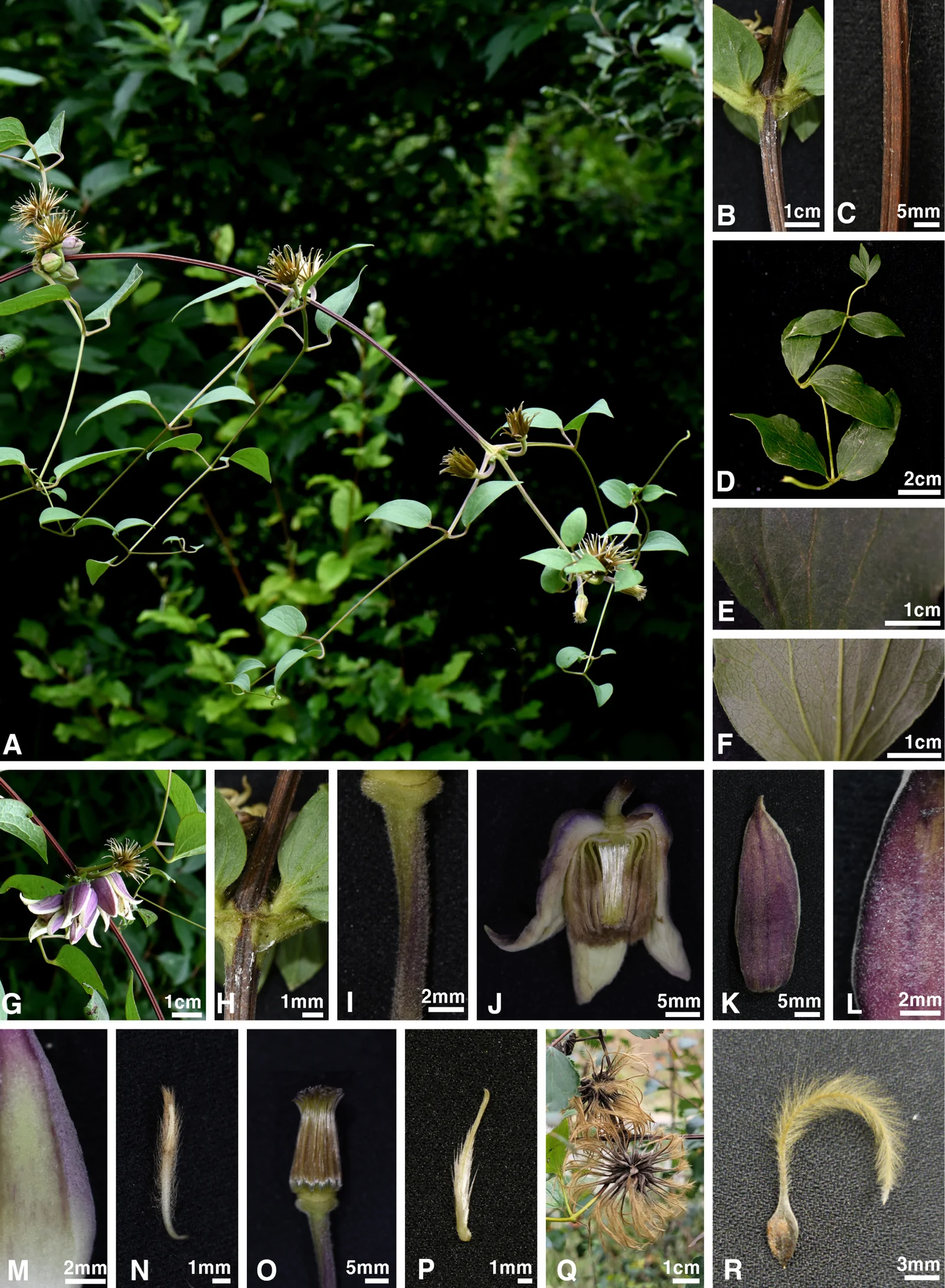
Photographs of *Clematis fusca* var. *violacea* Maxim. **A**. Habit; **B**. Basal portion of stem; **C**. Distal portion of stem; **D–F**. Leaves; **D**. General view of the leaf; **E**. Adaxial surface; **F**. Abaxial surface; **G**. Inflorescence; **H**. Peduncle; **I**. Pedicel; **J**. Bisexual flower; **K–M**. Sepals; **K**. General view of the sepal; **L**. Abaxial surface; **M**. Adaxial surface; **N**. Stamen; **O, P**. Pistils; **Q**. Aggregate of achenes; **R**. Achene.

*Clematis fusca* var. *violacea* differs from *C. fusca* var. *fusca* in having dingy violet sepals with sparsely white to yellowish brown pubescent to almost glabrous abaxial surfaces and densely whitish villous at the margins, whitish or yellowish brown pubescent pedicels, and climbing stems (Krasheninnikov 1970; Wang and Bartholomew 2001) (Fig. 7; Table 1).

##### Description.

Perennial herbaceous vines, hermaphroditic, scandent, reaching ca. 4 m in length. Stems grayish to dark brown, glabrate; nodes 3.2–8.5 mm diameter, villous; internodes 6.1–22.3 cm long, 1.7–4.3 mm diameter, distinctly grooved, white villous. Leaves opposite, simple or 3–7-foliolate, ternate or pinnate; petioles tendril-like, 2.8–7.9 cm long, 0.8–2.2 mm in diameter, yellowish green to reddish green, villous; petiolules tendril-like, yellowish green to reddish green, sparsely villous, terminal petiolule 0.5–3.9 cm long, 0.7–1.3 mm in diameter; lateral petiolules 0.5–4.2 cm long, 0.8–2.1 mm in diameter; leaflets ovate-lanceolate to widely ovate, apices acute to acuminate, margin entire and lobed, cleft or divided, base acute, rounded, or subcordate; adaxial surfaces yellowish green to dark green, sparsely pubescent; abaxial surfaces pale green, sparsely pubescent; terminal leaflets 1.1–7.3 × 0.5–6.2 cm; lateral leaflets 3.2–9.6 × 2.1–8.2 cm. Inflorescences cymes, terminal or axillary, 1–3-flowered. Bracts 1-paired or terminal flower absent, petiolate, 0.9–2.3 × 0.7–1.6 cm, lanceolate to widely ovate, entire to 3-lobed, yellowish green to green, pubescent. Peduncle inconspicuous, 0.9–6.7 mm long, green or reddish green, densely whitish or yellowish brown pubescent; pedicels 0.8–2.2 cm long, yellowish green or reddish green, whitish or yellowish brown pubescent. Flower bisexual, nodding, actinomorphic. Sepals 4, 1.8–3.3 cm × 6.8–14.7 mm, ovate to oblong-ovate, apices acuminate or acute, whitish violet to dark purple, adaxial sparsely white to yellowish brown pubescent and almost glabrous, abaxial sparsely white to yellowish brown pubescent to almost glabrous and densely whitish villous at margins. Stamens numerous; filaments 4.3–13.8 × 0.5–1.5 mm, white to yellowish white, brown villous at upper part; anthers 3.5–5.6 × 0.5–0.8 mm, linear, lanceolate or oblong, white to pale yellowish white, sparsely to densely brown villous at upper part. Pistils numerous. Ovaries 0.7–1.6 mm × 0.6–1.1 mm, ovoid or ellipsoid, yellowish green or green, white or yellowish brown villous; styles 6.4–16.6 mm long, green, densely yellowish brown to brown sericeous. Achenes 4.5–6.5 mm × 4.1–5.5 mm, flattened obovoid or ellipsoid, yellowish green or green, dark brown in maturity, white to yellowish brown villous; persistent styles 3.1–5.1 cm long, plumose, yellowish brown to brown sericeous.

##### Phenology.

Flowering from June to July and fruiting from July to September.

##### Etymology.

The varietal epithet “*violacea*” refers to the violet coloration of the sepals.

##### Korean vernacular name.

Jong-deong-gul (종덩굴).

##### Distribution and habitat.

*Clematis fusca* var. *violacea* is distributed in northeastern China, the Russian Far East, and Korea. In South Korea, it has been recorded only from Gyeonggi-do (Appendix 1). It occurs in inland forests and along forest margins.

##### Additional specimens examined.

See Appendix 1.

### Key to *Clematis pedunculata* and related Korean taxa

**Table:** 

1	Plants androdioecious; stem internodes smooth to indistinctly grooved, sparsely villous to glabrate; peduncle conspicuous, more than 1 cm long; stamens white pubescent distally	***C. pedunculata***
–	Plants hermaphroditic; stem internodes distinctly grooved, villous; peduncle almost absent, subsessile, or less than 7 mm long; stamens brown-villous distally	**2**
2	Sepal white to yellowish brown pubescent at abaxial surface	***C. fusca* var. *violacea***
–	Sepal dark brown to blackish brown tomentose at abaxial surface	**3**
3	Plants scandent, woody vines; petioles tendril-like; flowers terminal and axillary	***C. fusca* var. *fusca***
–	Plants erect, herbaceous or subshrub; petioles not tendril-like; flowers terminal only	***C. fusca* var. *flabellata***

## Discussion

The name *Clematis fusca* var. *violacea* has long been applied to Korean plants characterized by nodding purple flowers and predominantly whitish sepal hairs. Maximowicz (1859) emphasized violet sepals with whitish hairs largely confined to their margins in the protologue, and the variety was subsequently recognized in Korean taxonomic and floristic treatments (Lee 1996; Lee 2003; Lee 2006; Chang 2007; Kim 2017). Examination, however, revealed that Korean collections identified under this name comprise two morphologically coherent groups. Comparison with the protologue and available images of relevant specimens indicates that one group corresponds to *C. fusca* var. *violacea*, whereas the other is recognized here as *C. pedunculata*. Recognition of the new species therefore refines the circumscription of *C. fusca* var. *violacea* in Korea rather than excluding that variety from the Korean flora.

The longstanding confusion between the two taxa appears to have resulted from their similar overall floral morphology. Both have a scandent habit, nodding purple flowers, and predominantly whitish hairs along the sepal margins. Examination of flowering individuals and herbarium specimens confirmed that Korean plants corresponding to *C. fusca* var. *violacea* bear only bisexual flowers, whereas *C. pedunculata* is androdioecious, with individuals bearing either staminate or bisexual flowers. In addition to this difference in sexual system, *C. pedunculata* is distinguished by a consistent combination of vegetative and reproductive characters, including smooth to shallowly grooved and sparsely villous to glabrate stem internodes, conspicuous peduncles 1.5–12.1 cm long, and stamens bearing short whitish hairs distally (Fig. 1; Table 1). In contrast, *C. fusca* var. *violacea* has distinctly grooved and more densely villous stem internodes, subsessile or shortly pedunculate flowers, and brown-villous stamens distally. Floral sexuality, stem morphology, inflorescence structure, and floral indumentum have been regarded as taxonomically informative characters in the infrageneric classification and species delimitation of *Clematis* (Wang and Li 2005; Kadota 2006; Kim 2017). The concordant differentiation of these characters across living plants and herbarium specimens indicates that the two groups are separated by a morphological discontinuity rather than representing ordinary variation within a single variety.

The molecular analyses provide additional support for this morphological distinction (Figs 2, 3). In the ITS phylogeny, the three accessions of *C. pedunculata* formed a moderately supported clade distinct from the sampled Korean members of the *C. fusca* complex (UFBoot = 83%), although the deeper phylogenetic position of this clade was weakly resolved (Fig. 2). In the combined plastid phylogeny, the same accessions formed a moderately supported clade (UFBoot = 82%) distinct from all sampled Korean members of the complex (Fig. 3). The recovery of the three accessions as a cohesive lineage in both the nuclear and plastid analyses is congruent with the morphological evidence for the distinctness of *C. pedunculata*. However, these results do not identify the closest relative of the new species or establish its definitive infrageneric placement. Previous molecular studies have likewise shown that several deeper relationships and traditionally recognized groups within *Clematis* remain incompletely resolved and may be sensitive to taxon and marker sampling (Miikeda et al. 2006; Xie et al. 2011; Lehtonen et al. 2016). Broader taxon sampling within the *C. fusca* complex across northeastern Asia and the inclusion of additional molecular data will therefore be necessary to clarify the phylogenetic position of the new species.

The combined morphological and molecular evidence indicates that *C. pedunculata* is not merely an extreme morphological variant of Korean *C. fusca* var. *violacea* but a distinct species that remained unrecognized because of its superficial resemblance to that variety. This finding also demonstrates that sepal color and marginal indumentum alone are insufficient for identifying Korean plants previously assigned to *C. fusca* var. *violacea*. Reliable identification requires consideration of floral sexuality, peduncle development, the morphology and indumentum of the stem internodes, and the indumentum of the sepals and stamens. Verified records of *C. pedunculata* are currently restricted to South Korea, where the species occurs in inland forests, along forest margins, and occasionally along roadsides (Fig. 5). It is therefore provisionally regarded as endemic to South Korea. Nevertheless, specimens identified as *C. fusca* var. *violacea* from adjacent regions should be re-examined to determine whether *C. pedunculata* occurs elsewhere in northeastern Asia.

## Supplementary Material

XML Treatment for Clematis pedunculata

XML Treatment for Clematis fusca

## Appendix 1

**Specimens of Clematis pedunculata and related taxa examined in this study**

**1. *Clematis pedunculata* B.K.Park**

**KOREA. Gyeonggi-do**: Dongducheon-si, Mt. Soyo, 27 Jun. 1959, *W.T. Lee, s.n*. (KH) • 3 Jul. 2007, *W.K. Paik, Soyosan-070703-270* (KH) • Dongducheon-si, Mt. Soyo, 3 Jul. 2007, *G.Y. Chung, ANH-01080580* (KH) • Mt. Wangbang, 28 Jun. *1975, J.S. Cha, s.n*. (KH) • Mt. Wangbang, 28 Jun. 1975, *J.S. Cha, s.n*. (KH) • Mt. Wangbang, 10 Jul. 1976, *S.C. Ko, s.n*. (KH) • Gapyeong-gun, Cheongpyeong-myeon, 2 Jul. 1972, *W.T. Lee, s.n*. (KH) • Mt. Hwaya, 4 Jun. 1989, *G.J. Lee, s.n*. (KH) • Mt. Jugeum, 25 Jun. 2004, *E.S. Jeon, ESJeon-41243* (KH) • Gunpo-si, Mt. Suri, 28 Jun. 1975, *M.R. Jeong, s.n*. (KH) • Gwangju-si, Mt. Taehwa, 17 Jul. 1983, *H.C. Shin, s.n*. (KH) • Incheon-si, Mt. Cheonma, 4 Jul. 1998, *M.K. Seo, S02614* (KH) • Namyangju-si, Mt. Baekbong, 25 Jun. 1992, *O.H. Lee, s.n*. (KH) • Mt. Cheonma, 7 Jul. 1985, *J.O. Hyun, s.n*. (KH) • 7 Jul. 1985, *J.O. Hyun, s.n*. (KH) • 7 Jul. 1985, *J.O. Hyun, s.n*. (KH) • 21 Jul. 2005, *S.P. Hong et al., KHUS-20110249* (KH) • Paju-si, Jangneung, 28 Aug. 1965, *T.B. Lee et al., s.n*. (3 sheets, KH) • Pocheon-si, Gwangneung, 27 May 1986, *J.Y. Kim, s.n*. (KH) • 12 Jun. 1954, *T.G. Ju, s.n*. (KH) • 2 Jul. 1959, *T.H. Chung, s.n*. (2 sheets, KH) • 16 Jul. 1982, *N.S. Lee et al., s.n*. (KH) • 16 Jul. 1982, *S.H. Yeo, s.n*. (KH) • Aug. 1956, *I.M. Kim, s.n*. (KH) • 30 Aug. 1979, *I.M. Kim, s.n*. (10 sheets, KH) • Sep. 1958, *S.M. Park, s.n*. (4 sheets, KH) • 24 Sep. 1990, *J.Y. Kang, s.n*. (KH) • 10 Oct. 1986, *S.S. Kim, s.n*. (KH) • Gwangneung forest, 31 May 2024, *B.K. Park, Gwangneungsup-240531-001* (KH) • Korea National Arboretum, 10 Jun. 2024, *B.K. Park et al., Sumokwon-240610-001* (KH) • Korea National Arboretum, 10 Jun. 2024, *B.K. Park et al., Sumokwon-240610-002* (KH) • Mt. Jukyeop, 5 Aug. 2007, *G.Y. Chung, ANH-01080582* (KH) • Mulpurebong, 6 Jul. 2001, *S.H. Park and P.G. Lee, Gwang-10987* (KH) • Pyeongtaek-si, Mt. Deokam, 20 Jun. 1989, *S.H. Yeo and M.R. Han, s.n*. (KH) • Seoul-si, Anam-dong, 13 Jun. 1977, *G.H. Ko, s.n*. (KH) • Yangpyeong-gun, Mt. Biryong, 23 Jul. 2007, *B.U. Oh et al., Yangpyeonggun-070723-088* (KH) • Mt. Maebong, 10 Oct. 1959, *T.H. Chung, s.n*. (KH) • Mt. Sori, 14 Jul. 2009, *W.G. Paik, NAPI-1145* (KH) • Mt. Yongmun, 17 Jul. 1975, *S.J. Lee, s.n*. (3 sheets, KH). **Gangwon-do**: Chuncheon-si, Anbo-ri, 8 Aug. 1979, *W.T. Lee, s.n*. (KH) • Gangchon-ri, 28 Jun. 2011, *S.H. Cho and J.H. Lee, HHU-2011-0050* (3 sheets, KH) • 2 Sep. 2011, *G.H. Kim and J.H. Moon, G.H.Kim&J.H.Moon-0002* (KH) • Jeongjok-ri, 3 Jun. 1982, *W.T. Lee, s.n*. (KH) • Mt. Daeryong, 2 Jul. 2006, *S.H. Cho, YoungdongKim-2006-0743* (KH) • Mt. Yeonyeop, 20 Jul. 1997, *W.T. Lee, s.n*. (KH) • Hoengseong-gun, Mt. Bongbok, 22 Aug. 2009, *S.H. Cho et al., YoungdongKim-BB-2009-0144* (KH) • Mt. Chiak, 22 Jul. 2008, *S.C. Ko, Chiaksan-080722-021* (KH) • *HNHM-2009-0304* (KH) • *s.n*. (KH) • Mt. Obong, 30 Jul. 2008, *S.C. Ko, Obongsan-080730-062* (KH) • Mt. Seongji, 6 Jul. 2005, *E.S. Jeon, ESJeon-52034* (KH) • Yongso valley, 27 Sep. 2013, *Y.H. Hong et al., Hongcheongun(Gwangamri, Yongsogyegok-130927-049)* (KH) • Jeongseon-gun, Donggang river, 19 Jun. 2004, *J.M. Jeong and M.H. Park, 04060182* (4 sheets, KH) • Hwaam-myeon, 2 Jul. 2011, *H.G. Lee et al., 0802019* (KH) • Jeongseon-gun, Imgye-myeon, 17 Jul. 2011, *M.S. Choi and H.J. Yim, 0903091* (KH) • Pyeongchang-gun, Daegwanryeong, 23 Sep. 1975, *M.Y. Jo, s.n*. (3 sheets, KH) • Pyeongchang-gun, Geumdang valley, 22 Jul. 2009, *S.H. Park et al., Park-SH93096* (5 sheets, KH) • Gyechon-ri, 10 Jul. 2001, *W.S. Lee and C.K. Lee, HKim-676* (KH) • Mt. Bakji, 16 Jul. 2012, *C.H. Nam et al., L120769* (KH) • Mt. Balwang, 28 Jun. 2013, *D.C. Shin et al., KNU_BMBC_PH_0614-1* (KH) • 21 Jul. 2011, *D.C. Shin et al., Sumokwon-3157* (KH) • Mt. Geumdang, 29 Jun. 2008, *G.Y. Chung, ANH-01080166* (2 sheets, KH) • 18 Jul. 2012, *C.H. Nam et al., L120893* (KH) • 31 Aug. 2008, *G.Y. Chung et al., 080831-120* (KH) • 22 Jul. 2011, *D.C. Shin et al., Sumokwon-3223* (KH) • Mt. Gyebang, 15 Aug. 2010, *C.Y. Choe, An-C0803* (KH) • Taebaek-si, Mt. Taebaek, Jun. 1978, *I.M. Kim, s.n*. (11 sheets, KH) • Wonju-si, Mt. Chiak, 5 Jul. 1990, *Y.J. Jeon et al., s.n*. (2 sheets, KH) • 7 Jul. 1980, *T.B. Lee, s.n*. (KH) • 8 Jul. 2006, *K.O. Yoo, s.n*. (KH) • 15 Jul. 2008, *S.C. Ko, s.n*. (2 sheets, KH) • 29 Jul. 1975, *W.T. Lee, s.n*. (KH) • Surimji, 19 Sep. 1976, *W.T. Lee, s.n*. (2 sheets, KH) • Yeongwol-gun, Buk-myeon, 17 Jul. 2008, *J.O. Hyun and H.J. Gwon, NAPI-0418* (2 sheets, KH) • Neureupjae, 3 Jul. 2003, *J.O. Hyun et al., 2003061* (KH). **Chungcheongbuk-do**: Boeun-gun, Mt. Songni, 24 Jul. 1960, *T.B. Lee, s.n*. (KH) • Chungju-si, Chungju lake, 13 Jul. 2003, *B.U. Oh et al., Chungjusi-S5-030713-029* (KH) • Danyang-gun, Boguk temple, 20 Jun. 1964, *T.B. Lee et al., s.n*. (KH) • Mt. Sobaek, 4 Aug. 2008, *W.B. Cho, W.B.Cho-80817* (KH) • Yeongchun-myeon, 3 Jul. 2005, *B.U. Oh et al., Sobaeksan(Hyeongjebong)-050703-075~079* (KH) • Eumseong-gun, Mt. Gaseop, 6 Aug. 2014, *B.U. Oh et al., Eumseonggun-140806-063* (KH) • Goesan-gun, Geomseung-ri, 15 Aug. 1937, *B.S. Do, s.n*. (KH) • Mt. Songni, 27 Jun. 2013, *B.U. Oh et al., Goesangun-130627-184* (KH) • 27 Jun. 2013, *B.U. Oh et al., Goesangun-130627-004* (KH) • Jecheon-si, Bongyang-eup, 2 Jul. 2005, *B.U. Oh et al., Jecheonsi(Seokgiam)-050702-234~237* (KH) • Mt. Wolak, 14 Jun. 1988, *J.H. Lee, s.n*. (2 sheets, KH) • Seongo-ri, 28 Jun. 1991, *B.H. Choi, s.n*. (KH) • 28 Jun. 1991, *B.H. Choi, s.n*. (KH) • Songgye-ri, 18 Jun. 1964, *T.B. Lee et al., s.n*. (2 sheets, KH) • Okcheon-gun, Gwanmobong, 24 Jul. 2009, *S.C. Ko, HNHM-2009-1276* (KH) • 24 Jul. 2009, *S.C. Ko and H.J. Kim, s.n*. (KH). **Chungcheongnam-do**: Asan-si, Mt. Dogo, 30 Jul. 2006, *H.J. Choi et al., NGH60520* (KH) • Daejeon-si, Mt. Manin, 22 Jun. 2005, *W.G. Paik, P406* (KH) • Daejeon-si, Mt. Sikjang, 29 Jun. 1997, *K.H. Park, s.n*. (2 sheets, KH) • Gongju-si, Mt. Gyeryong, 3 Aug. 1957, *T.H. Chung, s.n*. (2 sheets, KH) • Seosan-si, Josan-si, 24 Aug. 1956, *T.H. Chung, s.n*. (KH). **Jeollabuk-do**: Buan-gun, Junggye-ri, 13 May 1981, *S.Y. Byun and Y.J. Wi, s.n*. (KH) • Ssangseonbong, 20 Jul. 2004, *G.Y. Chung, Buan-gun(Ssangseonbong, Junggye-ri)-040720-001* (KH) • Muju-gun, Mt. Jeoksang, 16 Jul. 2000, *B.Y. Sun and T.K. Baek, s.n*. (KH). **Jeollanam-do**: Gurye-gun, Banyabong, 5 Jul. 2013, *G.C. Chu et al., KNU_BMPC_PC_1403~1407* (KH) • Haenam-gun, Wolsong-ri, 18 Jul. 1958, *S.H. Park, s.n*. (KH) • Jangheung-gun, Mt. Suin, 2 Sep. 2003, *J.G. Ahn et al., s.n*. (2 sheets, KH) • Wondeung-ri, 21 Jul. 1995, *H.T. Im, Im-34752* (KH) • Mt. Naejang, 4 Aug. 1974, *Y.N. Lee and Y.J. Oh, s.n*. (2 sheets, KH) • Jangseong-gun, Mt. Naejang, 10 Aug. 1965, *J.H. Shin et al., s.n*. (KH) • Yeonggwang-gun, Mt. Bulgap, 29 May 1998, *D.O. Im and E.J. Yun, s.n*. (2 sheets, KH). **Gyeongsangbuk-do**: Andong-si, Mt. Yeonjeom, 4 Aug. 2006, *G.E. Yoo, Andongsi(Yeonjeonsan)-060804-094* (KH) • Cheongdo-gun, Mt. Hwaak, 2 Aug. 2006, *C.Y. Yoon, Yoon-578* (KH) • Cheongsong-gun, Gowa-ri, 18 Jun. 2024, *B.K. Park et al., Gowari-240618-001~003* (KH) • Cheongsong-gun, Sandubong, 28 Jun. 1998, *B.O. Oh, s.n*. (KH) • Cheongsong-gun, Sanjibong, 20 Jun. 2000, *S.W. Park et al., s.n*. (4 sheets, KH) • 9 Jul. 2000, *B.W. Lee et al., s.n*. (4 sheets, KH) • Cheongsong-gun, Surak-ri, 1 Jul. 2009, *M.H. Lee et al., s.n*. (KH) • Daegu-si, Mt. Palgong, 29 Jul. 1992, *s.c., s.n*. (KH) • Mungyeong-si, Mt. Dan, 12 Jul. 2008, *G.Y. Chung, ANH-01080246* (2 sheets, KH) • *G.Y. Chung et al., s.n*. (KH) • Mt. Noejeong, 1 Jun. 2002, *D.H. Choi et al., CBU13409* (KH) • *S.M. Kim et al., CBU13410* (KH) • *CBU13411* (KH) • 11 Jul. 2008, *G.Y. Chung, ANH-01080253* (KH) • Sangju-si, Mt. Bonghwang, 25 Jul. 2006, *G.Y. Chung, ANH-01060132* (2 sheets, KH) • Uiseong-gun, Binggye valley, 9 Jul. 2005, *J.S. Kim, kjs050592* (KH) • Geumhak-ri, 18 Jun. 2024, *B.K. Park et al., Geumhakri-240618-001* (KH) • Hupyeong-ri, 17 Jun. 1973, *S.Y. Oh, SOO-YOUNGOH005835* (KH) • Yeongcheon-si, Mt. Bohyeon, 7 Jul. 2000, *G.Y. Chung, ANH-01000024* (2 sheets, KH) • Yeongju-si, Sucheol-ri, 18 Jun. 2024, *B.K. Park et al., Sucheolri-240618-001* (KH). **Gyeongsangnam-do**: Changnyeong-gun, Mt. Hwawang, 22 Jul. 2016, *S.C. Ko et al., Hwawangsan-160722-016~18* (KH).

**2. *Clematis fusca* Turcz. var. *violacea* Maxim**.

**KOREA: Gyeonggi-do**: Ansan-si, Mt. Chilbo, 17 Jul. 2021, *B.K. Park, Chilbosan-210717-001* (2 sheets, KH) • Gapyeong-gun, Mt. Myeongji, 15 Aug. 1987, *S.S. Kim, Y.M. Lee, s.n*. (3 sheets, KH) • Namyangju-si, Mt. Baekbong, 2 Oct. 1979, *I.M. Kim, s.n*. (KH) • Pocheon-si, Gakheulbong, 25 Jun. 2008, *S.H. Oh, Gakheulbong-80625-81, 82* (KH) • Jikdong-ri, 27 Jul. 2005, *S.P.Hong et al., KHUS20110482* (KH) • 15 Jul. 2020, *B.K. Park et al., Sumokwon-200715-001, 002* (KH) • 11 Jul. 2022, *B.K. Park, Sumokwon-220711-001* (KH) • 26 Jun. 2023, *B.K. Park, Sumokwon-230626-001* (KH) • Gwangneung, Aug. 1956, *I.M. Kim, s.n*. (2 sheets, KH) • 11 Aug. 2000, *S.H. Park and Y.H. Kim, s.n*. (2 sheets, KH) • 24 Aug. 1986, *J.Y. Kim, s.n*. (KH) • Korea National Arboretum, 10 Jul. 2002, *J.S. Kim, s.n*. (KH) • 14 Jun. 2023, *B.K. Park, Sumokwon-230614-001* (KH) • Pyeonghwawon, 26 Jun. 2001, *E.S. Jeon and S.G. Gwon, s.n*. (3 sheets, KH) • 16 Jul. 2003, *S.H. Park, ParkSH-31643* (2 sheets, KH) • Sinheung-ri, 7 Jul. 2024, *K.H. Lee and D.H. Kim, Hantangang-240707-001* (KH).

**3. *Clematis fusca* Turcz**.

**KOREA: Gyeonggi-do**: Gapyeong-gun, Mt. Myeongji, 15 Aug. 1987, *S.S. Kim and Y.M. Lee, s.n*. (4 sheets, KH) • Pocheon-si, Gwangneung, 30 Aug. 1979, *I.M. Kim, s.n*. (KH). **Gangwon-do**: Donghae-si, Samhwa-dong, 26 Jul. 2011, *K.S. Lee et al., 248* (KH) • Gangneung-si, Mt. Seokbyeong, 18 Jun. 2006, *J.O. Hyun et al., 601022* (KH) • 20 May 2012, *H.T. Im, Im-090937* (KH) • Hoengseong-gun, Mt. Cheongtae, 26 Jul. 2008, *G.Y. Chung, ANH-01080174, 01080175, 01080284, 01080343* (KH) • 21 Jul. 2011, *G.Y. Byeon et al., HS110469* (KH) • 26 Aug. 2011, *G.Y. Byeon et al., N110782* (KH) • 19 Jul. 2019, *B.K. Park, Cheongtaesan-190719-001~034* (KH) • 19 Aug. 2019, *B.K. Park, Cheongtaesan-190819-001~009* (KH) • Hongcheon-gun, Mt. Eungbok, 16 Aug. 2013, *D.C. Shin and Balkrishna Sumokwon, 0880* (KH) • Inje-gun, Hyangrobong, 22 Jul. 2011, *H.G. Oh et al., Baekdudaegan-2011-074* (KH) • Mt. Daeam, 24 Jun. 2010, *Y.C. Kim, KYC-0698* (KH) • Mt. Daeam, 10 Jun. 2013, *H.J. Choi et al., 130610-hjchoi049, hjchoi050* (KH) • 29 May 2013, *S.H. Oh et al., KH1957* (KH) • 30 May 2013, *S.H. Oh et al., KH2080* (KH) • Mt. Jeombong, 26 Jun. 2013, *S.H. Park et al., P131601* (KH) • Jeongseon-gun, Mt. Baekun, 3 Jul. 2013, *H.T. Im and S.Y. Park, CNU-130211* (KH) • Mt. Seokbyeong, 21 Jun. 2013, *G.Y. Chung et al., ANH-04130115* (KH) • Pyeongchang-gun, Mt. Cheongtae, 29 Jun. 2008, *G.Y. Chung, ANH-01080174, 01080175* (2 sheets, KH) • 26 Jul. 2008, *G.Y. Chung, ANH-01080343, ANH-01080284* (2 sheets each, KH) • *G.Y. Chung et al., 080726-145* (KH) • Seonghwang temple, 12 Jun. 2012, *S.H. Park et al., ParkSH-121420* (KH) • Yongsan-ri, 1 Jul. 2013, *J.G. Ahn and E.H. Ra, JNU130701-50722* (KH) • Samcheok-si, Jungbong-ri, 6 Jun. 2011, *K.S. Lee et al., 419* (KH) • 26 Jul. 2011, *K.S. Lee et al., 432* (KH) • Mt. Cheongok, 14 Jun. 2006, *J.O. Hyun et al., 20060270* (KH) • Mt. Duta, 1 Jun. 2007, *J.O. Hyun et al., 701011* (KH) • 9 Jun. 2007, *J.O. Hyun et al., 601050* (KH) • Taebaek-si, Mt. Daedeok, 25 May 2012, *S.C. Ko et al., Taebaeksi(Changjukdong, Daedeok)-120525-293, 294*, (KB) • *S.C. Ko et al., Taebaeksi(Changjukdong, Daedeok)-120525-295, 296* (KH) • *H.S. Lee et al., HNHM-2012-0014* (KH) • Yanggu-gun, Mt. Daeam, 4 Jul. 2000, *C.S. Chang, s.n*. (KH) • Yanggu-gun, Mt. Dosol, 29 Jun. 2012, *K.O. Yoo et al., K.O.Yoo-0140* (KH) • 14 Aug. 2012, *K.O. Yoo et al., K.O.Yoo-0139* (KH) • 21 Jun. 2006, *S.H. Park and G.H. Nam, ParkSH-60942* (2 sheets, KH) • Yangyang-gun, Jobong, 30 May 2011, *D.C. Shin et al., Sumokwon-2692* (KH). **Gyeongsangbuk-do**: Bonghwa-gun, Okdolbong, 12 Jul. 2010, *W.C. Jeong and Balkrishna Sumokwon, 3674, 3675, 3676* (KH) • 19 Jul. 2010, *S.H. Oh et al., C100686* (KH) • Mt. Seondal, 13 Jul. 2010, *W.C. Jeong and Balkrishna Sumokwon, 3726, 3627* (KH).

**4. *Clematis fusca* Turcz. var. *flabellata* (Nakai) J.S.Kim**

**KOREA: Gangwon-do**: Gangneung-si, Daegwanryeong, 10 Jul. 2007, *H.G. Oh et al., Daegwanryeong-0003, 0051, 0122, 0239* (KH) • Mt. Gorupogi, 22 May 2008, *G.Y. Chung et al., 080522-384* (KH) • Mt. Odae, 3 Jun. 2004, *K.O. Yoo, s.n*. (KWNU) • Mt. Seokbyeong, 21 May 2006, *J.O. Hyun et al., 401024, 402032* (KH) • 4 Jun. 2006, *J.O. Hyun et al., 501017, 502005* (KH) • Noinbong, 16 Jun. 2004, *K.O. Yoo, s.n*. (KWNU) • 9 Sep. 2006, *E.S. Jeon, ESJeon-61496* (KH) • Sangye-ri, 5 Jun. 2011, *J.D. Lee, PWK-045* (KH) • Goseong-gun, Hyangrobong, 29 Jul. 2010, *K.O. Yoo, s.n*. (KWNU) • Mt. Ma, 17 May 2008, *W.K. Paik, HNHM-2009-1274, 1275* (KH) • Hoengseong-gun, Bugok-ri, Mt. Chiak, 22 Jul. 2008, *S.C. Ko, s.n*. (HNHM) • Hongcheon-gun, Bangnae-ri, 22 Jun. 2005, *J.S. Kim, kjs050668* (KH) • Hongcheon-gun, Nae-myeon, 16 Jul. 2005, *E.S. Jeon, ESJeon-52279* (KH) • Hongcheon-gun, Solchijae, 10 May 2006, *E.S. Jeon, ESJeon-60274* (KH) • Hwacheon-gun, Mt. Il, 23 May 2008, *K.O. Yoo, s.n*. (KWNU) • 29 May 2008, *B.H. An, Ilsan-80529-120, 122, 132, 141, 164* (KH) • 27 May 2010, *K.O. Yoo, s.n*. (KWNU) • Inje-gun, Daeseungryeong, 21 May 1997, *Y.M. Lee, S-4106, 1669* (KH) • Danmoknyeong, 3 Jun. 2010, *K.O. Yoo, s.n*. (KWNU) • Hyangrobong, 7 Jun. 2012, *J.S. Shim and C.H. Lee, GW_V2012019* (KH) • Jochimnyeong, 22 May 2010, *K.O. Yoo, s.n*. (KWNU) • Mt. Jeombong, 21 Jun. 2007, *J.S. Park et al., Kss1088, Kss1089* (KH) • Mt. Seorak, 2 Sep. 2004, *J.M. Jeong and J.Y. Kim, 04090012* (KH) • 8 Aug. 2013, *G.Y. Chung et al., ANH-04130234, ANH-04130272* (KH) • Jeongseon-gun, Duwibong, 25 Jun. 2008, *S.H. Oh, Duwibong-80625-41* (KH) • Eundaebong, 30 May 2019, *B.K. Park, Eundaebonng-190530-001~005* (KH) • 18 Aug. 2019, *B.K. Park, Eundaebonng-190818-001* (KH) • Geumdaebong, 17 Jul. 2003, *E.S. Jeon, ESJeon-31258* (KH) • 24 May 2005, *E.S. Jeon, ESJeon-51192* (2 sheets, KH) • 6 Jun. 2006, *G.Y. Chung et al., ANH-04060012* (KH) • 30 May 2019, *B.K. Park, Geumdaebonng-190530-001~004* (KH) • 18 Aug. 2019, *B.K. Park, Geumdaebonng-190818-001~004* (KH) • Mt. Hambaek, 12 Aug. 1999, *W.T. Lee, s.n*. (KWNU) • 9 Aug. 2009, *S.C. Ko, HNHM-2009-1272, HNHM-2009-1273* (KH) • 9 May 2010, *E.S. Jeon, ESJeon-100535* (KH) • 18 May 2011, *C.H. Lee et al., HS110031* (KH) • 28 Jun. 2011, *C.H. Lee et al., BK110017* (KH) • 28 Jun. 2011, *S.Y. Jeong and H.S. Hwang, JSY110013* (KH) • 29 Jun. 2011, *J.C. Yang et al., JJ110063* (KH) • 29 Jun. 2011, *S.J. Ji et al., JSY110112* (KH) • 1 Jul. 2015, *S.J. Ji and J.S. Han, KH150127* (KH) • Mt. Gariwang, 29 May 2019, *B.K. Park, Gariwangsan-190529-014~019* (KH) • Pyeongchang-gun, Daegwanryeong, 29 Jun. 2010, *S.H. Park and H.S. Choi, ParkSH-101289* (KH) • Hoenggye2-ri, 20 Jul. 2009, *S.-H. Park et al., ParkSH-92946* (KH) • Mt. Bakji, 28 Jun. 2008, *G.Y. Chung et al., ANH-04120114* (KH) • 25 May 2008, *G.Y. Chung, ANH-01080221* (2 sheets, KH) • 24 May 2012, *J.C. Yang et al., H120128* (KH) • Mt. Cheongok, 30 Jun. 2008, *G.Y. Chung, ANH-01080177* (2 sheets, KH) • 24 May 2008, *G.Y. Chung, ANH-01080202* (2 sheets, KH) • Mt. Gorupogi, 22 May 2008, *G.Y. Chung, ANH-01080155* (2 sheets, KH) • Mt. Hwangbyeong, 27 Jul. 2008, *G.Y. Chung, ANH-01080514* (2 sheets, KH) • Mt. Odae, 30 Jun. 2008, *G.Y. Chung, ANH-01080184* (2 sheets, KH) • Neunggyeongbong, 13 Jun. 2006, *J.O. Hyun et al., 20060144, 20060145* (KH) • Samyang ranch, 20 Jul. 2009, *S.H. Park et al., ParkSH-92946* (4 sheets, KH) • SeonJaryeong, 29 Jun. 2010, *S.H. Park and H.S. Choi, ParkSH-101229* (2 sheets, KH) • Yongsan-ri, 4 Jun. 2011, *J.D. Lee, PWK-021* (KH) • Samcheok-si, Jungbong-ri, 6 Jun. 2011, *K.S. Lee et al., 299* (KH) • Mt. Duta, 9 Jun. 2007, *J.O. Hyun et al., 601047* (KH) • Mt. Duta, 1 Jul. 2007, *J.O. Hyun et al., 701005* (KH) • Sokcho-si, Hwangcheolbong, 21 May 2008, *W.K. Paik, PWK-0305* (KH) • 10 Jul. 2008, *W.K. Paik, Hwangcheolbong-080710-145* (KH) • Mt. Seorak, 5 Jun. 2013, *D.K. Kim and K.H. Oh, 2013V029* (KH) • Taebaek-si, Geumdaebong, 6 Jun. 2006, *G.Y. Chung, ANH-01060046* (2 sheets, KH) • 6 Sep. 2011, *G.-C. Lee et al., 2011113* (KH) • 4 Jun. 2009, *J.H. Kim et al., 2009032* (KH) • Mt. Hambaek, 26 May 1998, *S.-S. Kim & Y.-M. Lee, S-1671, S-1672* (KH) • 1 Sep. 2015, *S.J. Ji et al., HJ150065* (KH) • Wonju-si, Mt. Chiak, 15 Jul. 2008, *S.C. Ko, s.n*. (2 sheets, HNHM) • Yanggu-gun, Mt. Daeam, 13 May 2000, *C.S. Chang, s.n*. (KH) • 22 May 2003, *E.S. Jeon, ESJeon-30182* (KH) • 22 Jun. 2006, *S.H. Park and G.H. Nam, ParkSH-60869* (4 sheets, KH) • Mt. Dosol, 21 Jun. 2006, *S.H. Park and G.H. Nam, ParkSH-60963* (5 sheets, KH) • 16 Jun. 2009, *S.S. Kim, Kim-98-7* (KH) • Yangyang-gun, Mt. Seorak, 20 Aug. 2008, *W.K. Paik, PWK-0319* (KH). **Gyeongsangbuk-do**: Bonghwa-gun, Mt. Seondal, W.C. Jeong and Balkrishna, 13 Jul. 2010, *Sumokwon, 3732, 3733* (KH).

## Additional information

### Artificial Intelligence (AI) use

The authors accept full responsibility for the content of the manuscript, including the disclosure of any use of AI.

No AI tools were used in the preparation of this manuscript.

### Author ORCIDs

B.K. Park https://orcid.org/0000-0003-1894-3296

D.-H. Lee https://orcid.org/0009-0008-5793-6893

S.-C. Kim https://orcid.org/0000-0003-0698-1500

D.C. Son https://orcid.org/0000-0002-6773-0580

### Data availability

All of the data that support the findings of this study are available in the main text.
